# Celf1 Is Required for Formation of Endoderm-Derived Organs in Zebrafish

**DOI:** 10.3390/ijms140918009

**Published:** 2013-09-03

**Authors:** Naoyuki Tahara, Yasumasa Bessho, Takaaki Matsui

**Affiliations:** Gene Regulation Research, Nara Institute Science and Technology, 8916-5 Takayama, Nara 630-0101, Japan; E-Mails: n-tahara@bs.naist.jp (N.T.); ybessho@bs.naist.jp (Y.B.)

**Keywords:** post-transcriptional regulation, endoderm, liver, pancreas, movement, proliferation

## Abstract

We recently reported that an RNA binding protein called Cugbp Elav-like family member 1 (Celf1) regulates somite symmetry and left-right patterning in zebrafish. In this report, we show additional roles of Celf1 in zebrafish organogenesis. When *celf1* is knocked down by using an antisense morpholino oligonucleotides (MO), liver buds fail to form, and pancreas buds do not form a cluster, suggesting earlier defects in endoderm organogenesis. As expected, we found failures in endoderm cell growth and migration during gastrulation in embryos injected with *celf1*-MOs. RNA immunoprecipitation revealed that Celf1 binds to *gata5* and *cdc42* mRNAs which are known to be involved in cell growth and migration, respectively. Our results therefore suggest that Celf1 regulates proper organogenesis of endoderm-derived tissues by regulating the expression of such targets.

## 1. Introduction

Cugbp Elav-like family member 1 (Celf1), which is a member of the Celf family of RNA binding proteins, regulates gene expression at multiple post-transcriptional levels including alternative splicing and mRNA decay, and fine-tunes the amounts of proteins that are synthesized from its target mRNAs [1–3]. Celf1 target mRNAs have been identified by several approaches such as the yeast three hybrid system [4], systematic evolution of ligands by exponential enrichment (SELEX) [5], RNA immunoprecipitation followed by microarray (RIP-Chip), and cross-linking immunoprecipitation followed by sequencing (CLIP-Seq) [6–10]. Celf1 binds to hundreds of short-lived mRNAs, which are involved in cell growth, migration and death [7]. On the basis of these analyses, UG-rich or UGU repeats are identified as a Celf1 binding sequences [3,7].

It has been reported that Celf1 is involved in the regulation of somite segmentation, muscle formation, and spermatogenesis in vertebrate development [6,11,12]. In addition, we recently showed that *celf1* is required for somite symmetry and cardiac laterality [13]. In the course of our investigation of other laterality organs such as liver and pancreas, we find an unexpected role for *celf1* in zebrafish organogenesis; *celf1* is essential for the formation of endoderm-derived organs.

## 2. Results and Discussion

### 2.1. *Celf1* Is Involved in Formation of Endoderm-Derived Organs

**Figure 1:**
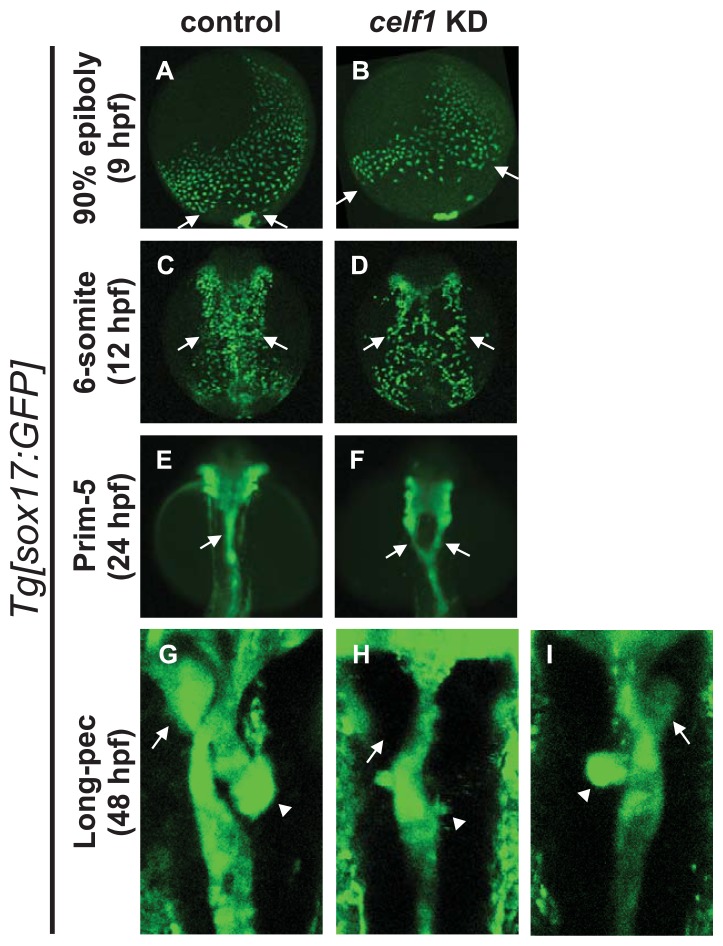
Knockdown of *celf1* leads to defects in endoderm-derived organs. (**A**–**D**) Lateral views of *Tg* [*sox17:GFP*] transgenic embryos at 9 hpf (**A**,**B**). Dorsal views of the mid-trunk region of *Tg* [*sox17:GFP*] transgenic embryos at 12 hpf (**C**,**D**). Anterior to the top. The migration of GFP-expressing endoderm cells to the dorsal midline was delayed in *celf1* KD embryos. Arrows in panels A, B and C, D point at the caudal and lateral edges of endoderm cells, respectively. Panels A and B are frames of supplementary movies 1 and 2, respectively. (**E**,**F**) Dorsal views of the pharyngeal and foregut regions of *Tg* [*sox17:GFP*] transgenic embryos at 24 hpf. Anterior to the top. *celf1* KD embryos showed a splitting of the anterior gut (arrow). (**G**–**I**) Dorsal views of the mid-trunk region of *Tg* [*sox17:GFP*] transgenic embryos at 48 hpf. Anterior to the top. Signs of liver buds (arrows) and pancreas buds (arrowheads) were lost or lower. *celf1* KD embryos resulted in defects of endoderm-derived organs (**H**,**I**) and left-right patterning (**I**).

Since cardiac laterality was altered in *celf1* knockdown (KD) embryos [13], we reasoned that the lateralities of endoderm-derived organs such as liver, pancreas and gut are also altered in *celf1* KD embryos. To test this, we injected *celf1* morpholinos (*celf1*-MOs) into transgenic line *Tg* [*sox17:GFP*] [14], whose GFP is expressed in endoderm cells and dorsal forerunner cells, and observed the formation of endoderm-derived organs in *celf1* KD embryos. In control embryos at 48 h postfertilization (hpf), liver and pancreas were formed on the left and right sides of the gut tube, and this tube was bent because the placement of these organs and looping are regulated by left-right patterning (Figure 1G) [15–17]. However, in the *celf1* KD embryos, signs of the liver bud, pancreas buds were low, and the gut tube tended to be straight (Figure 1H). Although we could observe left-right defects in the gut tube in the *celf1* KD embryos (Figure 1I), other defects were unexpected. These results therefore suggest that *celf1* has an additional role(s) in the formation of endoderm-derived organs in zebrafish.

**Figure 2:**
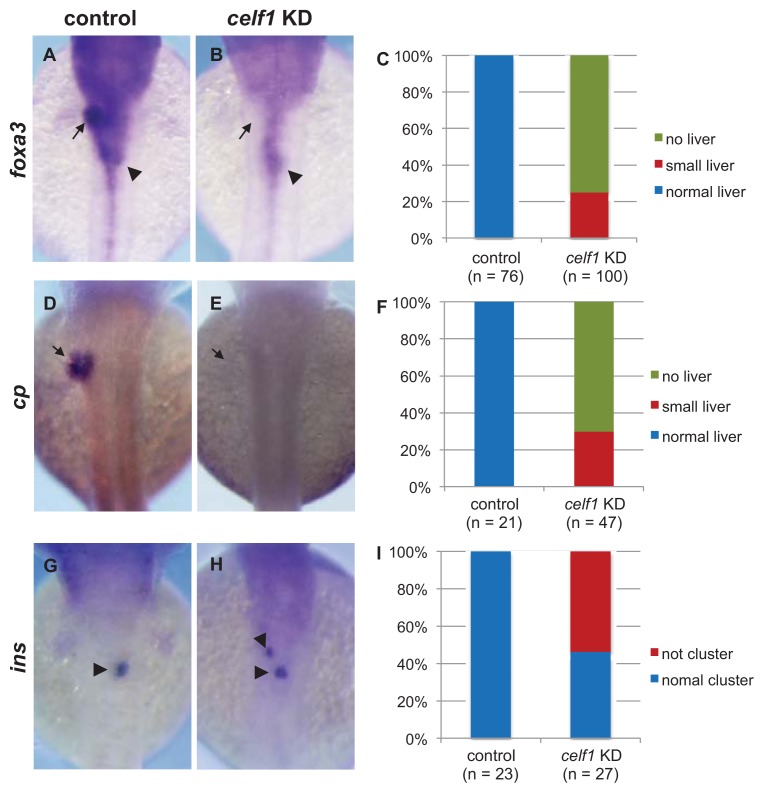
Malformations of endoderm-derived organs in *celf1* KD embryos. The expression of *foxa3* (gut and its associated organs), *cp* (liver), and *ins* (β-cells in pancreas) was examined in *celf1* KD embryos at 48 hpf. Expression of *foxa3* was specifically lost at the anterior part of the foregut in *celf1* KD embryos (**A**–**C**). Moreover, the liver buds did not form (arrows, **D**–**F**), and β-cells in the pancreas bud did not form a cluster (arrowheads, **G**–**I**) in the *celf1* KD embryos.

To investigate the role of *celf1* in the formation of endoderm-derived organs, we analyzed the expression of markers for general endoderm derivatives (*forkhead box A3*, *foxa3*), liver fate (*ceruloplasmin*, *cp*), and pancreas differentiation (*preproinsulin*, *ins*). Consistent with the results seen in the *Tg* [*sox17:GFP*] embryos, liver buds became smaller or absent in the *celf1* KD embryos (Figure 2A–F). Although β-cells in the pancreas formed a cluster by 48 hpf in the control embryos, two or three populations of β-cells were visible in the *celf1* KD embryos (Figure 2G–I). These results suggest that *celf1* is essential for the formation of endoderm-derived organs.

### 2.2. *Celf1* Controls Endoderm Cell Growth during Gastrulation

**Figure A1:**
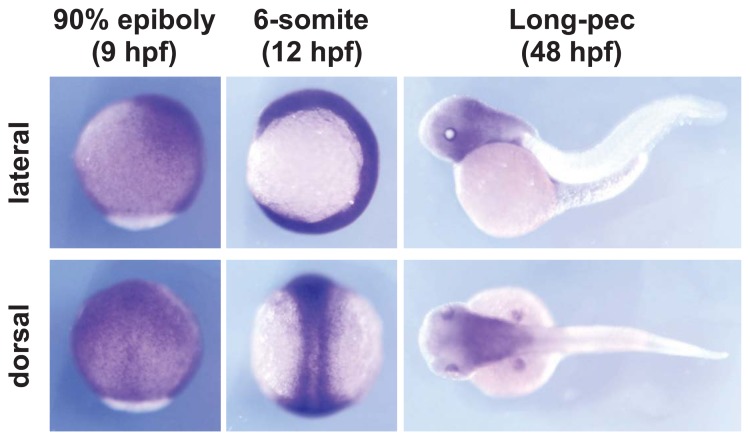
*celf1* expression in zebrafish embryos. Lateral (**upper panels**) and dorsal views (**lower panels**). We could observe dotted signals at 9 hpf (**left panels**), reminiscent for the distribution of endoderm cells in this stage (see also Figure 1A). Although endoderm cells distributed in a salt and pepper pattern at 12 hpf as shown in Figure 1C, the salt and pepper distribution of *celf1* could not be seen in embryos at 12 hpf. At 48 hpf, *celf1* was expressed in pectoral fins, heads and eyes, but not in endoderm-derived organs. Because mouse *Celf1* and chick *Celf1* are expressed in livers [26–30], we think that the expression patterns of *celf1* are specific for zebrafish.

As a maternal factor, *celf1* is broadly expressed in zebrafish embryos by gastrulation stages, whereas *celf1* expression is not detected in endoderm derivatives (Figure A1). Instead, *celf1* expression is restricted to specific regions such as eyes and pectoral fins at later stages [13,18,19]. We therefore reasoned that *celf1* regulates endoderm formation during gastrulation and secondarily affects the formation of endoderm-derived organs in later embryos. To test the possibility, we observed the behavior of endoderm cells during gastrulation by using *Tg* [*sox17:GFP*] embryos. GFP-expressing endoderm cells were distributed around the blastoderm margin in a salt-and-pepper pattern in control embryos at 6 hpf and they then migrated dorsally and proliferated (Movie 1). Although endoderm cells appeared normally in *celf1* KD embryos at 6 hpf, dorsal migration of endoderm cells became slow and the number of the cells seemed to be low in comparison with the controls (Figure 1A,B, and Movies 1 and 2).

**Figure 3:**
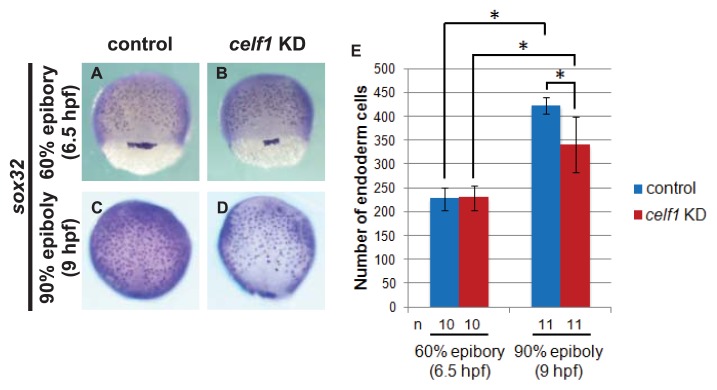
*celf1* controls endoderm cell growth during gastrulation. (**A**–**D**) *sox32* expression in endoderm cells at 6.5 and 9 hpf in control and *celf1* KD embryos. Anterior to the top. (**E**) The number of endoderm cells was the same at 6.5 hpf but reduced at 9 hpf. Asterisks indicate statistically significant differences (* *p* < 0.05).

**Figure A2:**
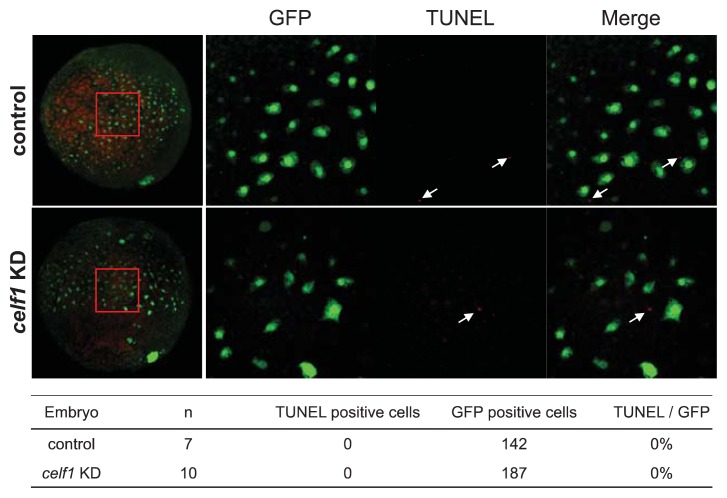
Cell death in zebrafish embryos at 90% epiboly stage. TUNEL assays were performed in *Tg* [*sox17:GFP*] embryos at 9 hpf. Green and red signals indicated GFP-expressing endoderm cells and TUNEL positive cells, respectively. Arrows marked TUNEL positive cells. Right panels were higher magnification images highlighting endoderm cells with the embryo (**left panel**). TUNEL signals were detected within the embryos, but we could not find TUNEL positive endoderm cells in control and *celf1* KD embryos.

**Figure A3:**
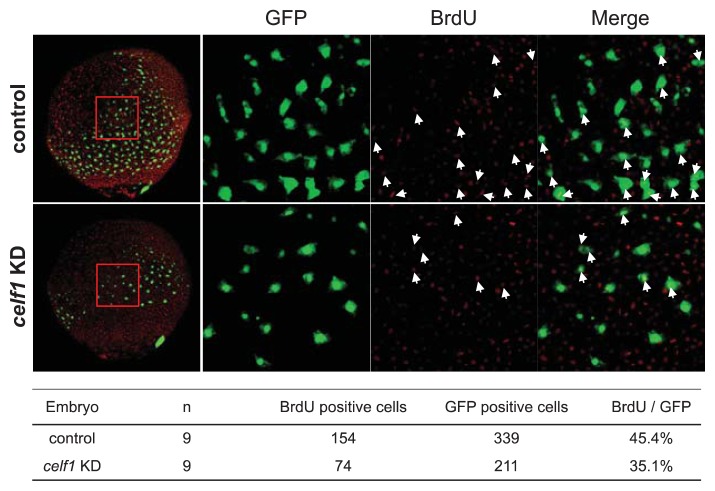
Cell proliferation in zebrafish embryos during gastrulation. BrdU staining was performed in *Tg* [*sox17:GFP*] embryos at 9 hpf. Green and red signals indicated GFP-expressing endoderm cells and BrdU positive cells, respectively. Right panels were higher magnification images highlighting endoderm cells with the embryo (**left panel**). The number of BrdU positive endoderm cells (arrows, double positive cells) significantly decreased in *celf1* KD embryos (*p < 0.05).

To confirm whether the endoderm cell number is reduced in the *celf1* KD embryos, we analyzed the expression of an endoderm specification marker (*sox32*) and counted the number of *sox32*-expressing endoderm cells. The number of cells in the *celf1* KD embryos was normal at 6 hpf but became significantly lower at 9 hpf (Figure 3). These results suggest that, in *celf1* KD embryos, endoderm specification occurs normally, but proliferation and/or death of endoderm cells are altered. We thus tested whether *celf1* regulates endoderm cell death, growth or both during gastrulation. Fragmented GFP signals, which are a sign of dead cells [20], were not observed both in the control and *celf1* KD embryos during the dorsal migration of the endoderm cells (Table 1). This result was supported by the data from TUNEL assays (Figure A2). In contrast, the number of cell divisions became significantly lower in the *celf1* KD embryos (*p* < 0.05, Table 1 and Movies 1 and 2). In agreement with this, BrdU incorporation of endoderm cells in *celf1* KD embryos significantly reduced as compared to that of control embryos (*p* < 0.05, Figure A3). These results suggest that *celf1* regulates endoderm proliferation during gastrulation.

### 2.3. *Celf1* Regulates Endoderm Cell Migration during Gastrulation

**Figure 4:**
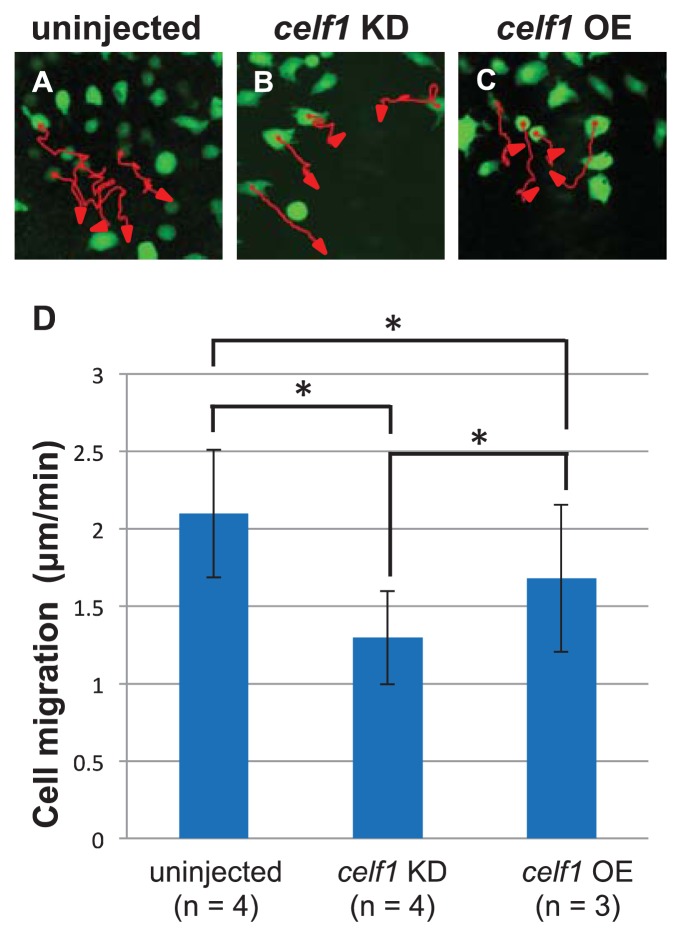
*celf1* regulates migration of endoderm cells during gastrulation. (**A**–**C**) The migration tracks individual endoderm cells in a period of 80 min in uninjected (**A**), *celf1* KD (**B**) or *celf1* overexpressing (OE) embryos (**C**). The cell position was determined every two minutes. Each red arrow indicates the trajectory of the cell migration. (**D**) Migration of endoderm cells (*n* = 58, 59 or 50) in uninjected (*n* = 4), *celf1* KD (*n* = 4) or *celf1* OE embryos (*n* = 3). The speed of cell migration in *celf1* KD embryos became slower than that of the uninjected control. Statistically significant differences (* *p* < 0.05) could be seen in control *vs. celf1* KD embryos.

Since time lapse observations suggest that cell migration is also limited in *celf1* KD embryos, we next investigated endoderm cell movements during gastrulation by tracing the trajectory of each GFP-expressing endoderm cell. In the *celf1* KD embryos, many endoderm cells migrated toward the midline, but the speed of the migration became slower than that of the uninjected control, leading to a defect in endoderm cell assembly around the midline (Figure 4). Consistent with the defect in endoderm migration during gastrulation, distribution of endoderm cells in the *celf1* KD embryos at 12 hpf became wider in comparison with that of the control (Figure 1C,D). Knockdown of *celf1* then resulted in a failure to fuse the anterior gut tube, leading to formation of a Y-shaped tube (Figure 1E,F). With the data taken all together, our results suggest that *celf1* regulates the growth and migration of endoderm cells during gastrulation to generate endoderm-derived organs properly.

### 2.4. *Celf1* Binds to *gata5* and *cdc42* mRNAs *In Vivo*

**Figure 5:**
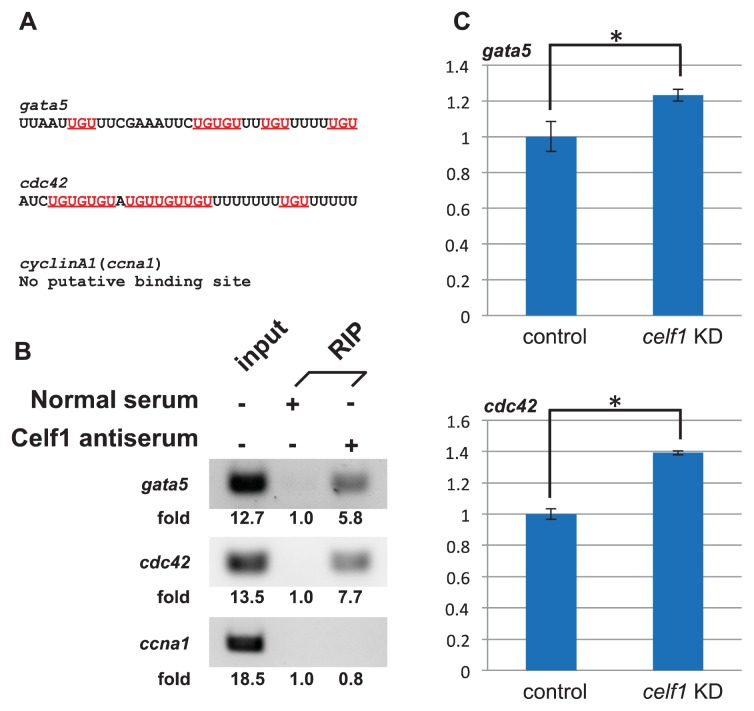
Celf1 binds to several mRNAs which encodes regulators of cell growth and migration in zebrafish embryos. (**A**) Putative Celf1 binding sequences within 3′ UTR of candidate mRNAs. Underlines mark UGU repeats. (**B**) Binding of Celf1 with specific RNA was tested with RIP assays. Celf1 associated with *gata5 and cdc42* mRNAs but not with *ccna1* mRNA (negative control). Signal intensities ware quantified by the Image J software. The signal intensity of each RIP sample by normal serum was stated as a basal level (1.0). (**C**) Effect of Celf1 on expression of *gata5* and *cdc42* in zebrafish embryos. Total RNAs extracted from embryos injected with control-MO or *celf1*-MOs at 10 hpf were subjected to qPCR for *gata5*, *cdc42* and *ccna1*. The samples were normalized to *ccna1* as a reference. Asterisks indicate statistically significant differences (* *p* < 0.05).

Celf1 binds to UG-rich elements or UGU repeats within the 3′ untranslated regions (3′ UTR) of mRNAs and regulates gene expression at multiple post-transcriptional levels [1–3]. Since *celf1* is essential for the growth and migration of endoderm cells, we looked for possible targets by undertaking a search for genes involved in endoderm proliferation and migration. Because either UG rich sequences or UGU repeats are existed in 3′ UTR of many mRNAs that encode cell cycle and migration regulators [5] and because flanking U-rich or UA-rich elements to UG/UGU sequences also affects the binding affinity of Celf1 [13], we thought that a sequence containing U/UA-rich elements and at least four UGU repeats within 35 bp would be a strong candidate for the Celf1-binding site. We thus selected *gata5* (*gata-binding protein 5*) and *cdc42* as potential targets of Celf1 for the following reasons (Figure 5A). *gata5* is known to control endoderm proliferation in zebrafish [21,22]. Both five UGU repeats and U-rich elements are present in 3′ UTR of *gata5* mRNA (Figure 5A). Rho family G proteins (Rho, Rac, Cdc42) regulate the convergence extension (CE) movements of mesoendoderm [23], but Rac and Cdc42 (but not Rho) control primordial midgut cells in the fly [24]. Among Rho family G proteins, only *cdc42* mRNA carries a putative Celf1 binding site that is composed of seven UGU repeats and U-rich sequences (Figure 5A). In addition, *Gata5* and/or *Cdc42* are identified as putative Celf1 targets in mouse muscle cells [6] and human T cells [8]. To test whether Celf1 binds to *gata5* and *cdc42* mRNAs *in vivo*, we performed RIP assays by using Celf1 antiserum. Although Celf1 did not bind to *cyclinA1* (*ccna1*) mRNA (negative control) [13], Celf1 associated with *gata5* and *cdc42* mRNAs (Figure 5B). To investigate whether Celf1 affects the expression of *gata5* and *cdc42*, we performed qPCR analyses in control and *celf1* KD embryos. Knockdown of *celf1* resulted in a 23% and 39% increase of the amounts of *gata5* and *cdc42* mRNAs relative to control, respectively (Figure 5C). These results suggest that Celf1 controls the formation of endoderm-derived organs through modulating protein expression from such targets during zebrafish development.

### 2.5. *Celf1* Targets

**Figure A4:**
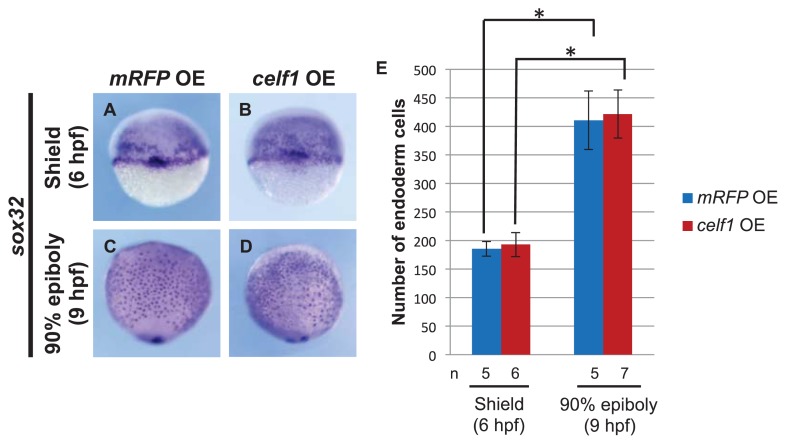
Effect of *celf1* overexpression on endoderm proliferation. (**A**–**D**) *sox32* expression in endoderm cells at 6 (**A**,**B**) and 9 hpf (**C**,**D**) in *mRFP* OE (**A**,**C**) and *celf1* OE embryos (**B**,**D**). Anterior to the top. (**E**) Overexpression of *celf1* did not affect endoderm proliferation. Asterisks indicate statistically significant differences (* *p* < 0.05).

Because loss-of-function of *gata5* resulted in the reduction of endoderm proliferation [21,22], we expected that Celf1 stabilize *gata5* mRNA. However, we got opposite results from qPCR analyses: Celf1 may destabilize *gata5* mRNA (Figure 5C). In addition, overexpression of *celf1* did not affect endoderm proliferation (Figure A4). Although Celf1 controls the levels of *gata5* mRNA, our and previous observations suggest that endoderm proliferation is regulated by complicated mechanisms, to which several factors contribute. To control the CE movements during gastrulation, Cdc42 is activated by non-canonical Wnt signaling [23]. Consistent with the fact that both loss- and gain-of-functions of the signaling showed CE defects [23], knockdown and overexpression of *celf1* resulted in slower migration of endoderm cells relative to uninjected control samples (Figure 4). Thus, *cdc42* is a strong candidate of the target to regulate endoderm migration.

However, we could not conclude yet whether these interactions are sufficient for controlling endoderm proliferation and migration. As reported previously [13], blocking the interaction of Celf1 with either *gata5* or *cdc42* mRNA using specific target protector morpholinos will be required. Because UG-rich elements or UGU repeats are present in numerous mRNAs, it is also possible that Celf1 coordinates protein expression from several targets to generate endoderm-derived organs properly. Therefore, systematic analyses including cross-linking RNA immunoprecipitation followed by microarray or sequencing will be important for understanding all of the roles of Celf1 in endoderm formation.

### 2.6. Roles of *Celf1* in Generation of Endoderm-Derived Organs

Our data suggest that Celf1 regulates endoderm formation during gastrulation. However, in *celf1* KD embryos at later stages, we could find several failures such as defective convergence of the gut tube, loss of the liver, and malformation of the pancreas (Figures 1 and 2). Although it is possible that these failures are secondary defects of endoderm formation at an earlier stage, one possibility is that Celf1 also contributes to generating endoderm-derived organs in later stages. Since *celf1* is not expressed in endoderm-derived organs in later stages (Figure A1), it is suggested that *celf1* non-cell autonomously affects the formation of endoderm-derived organs. It would be of great interest to prove the stage and cell type specific roles of Celf1 in zebrafish embryos.

## 3. Experimental Section

### 3.1. Zebrafish and Whole-Mount *In Situ* Hybridization

Wild-type and *Tg* [*sox17:GFP*] [14] zebrafish were used in this study. Whole-mount *in situ* hybridization was performed as described previously [20,25]. cDNA fragments of *celf1*, *cp*, *foxa3*, *ins*, and *sox32* were used as templates for the antisense probes.

### 3.2. Morpholino and mRNA Injection

Antisense MO oligonucleotides named *celf1_long-*MO, *celf1_short*-MO, and control-MO were obtained from Gene Tools. *celf1_long-*MO and *celf1_short*-MO were designed to target the AUG initiation codon of these mRNAs.

The MO sequences were as follows:

control-MO: 5′-CCTCTTACCTCAGTTACAATTTATA-3′;
*celf1_long*-MO: 5′-GCTTCAGCTTCGATACTATCCATCC-3′ [13];
*celf1_short*-MO: 5′-GTGGTCCAGAGACCCATTCATCTTC-3′ [13].

To knock down *celf1*, we co-injected 2.5 ng *celf1_long*-MO and 2.5 ng *celf1_short*-MO (5 ng *celf1*-MOs) into one-cell-stage zebrafish embryos. As a control, we injected 5 ng control-MO. We previously evaluated the specificity and efficacy of *celf1_long*-MO and *celf1_short*-MO [13].

pCS2-*celf1* (long form) and pCS2-*monomeric red fluorescent protein* (*mRFP*) were used in this study. *celf1* and *mRFP* mRNAs were synthesized using SP mMassage mMachine System (Ambion, Carlsbad, CA, USA). To overexpress *celf1*, we injected 150 pg *celf1* mRNA into one-cell-stage embryos. As a control, 150 pg mRFP mRNA was injected.

### 3.3. TUNEL and Immunofluorescence Analyses

*Tg* [*sox17:GFP*] embryos at 90% epiboly stage (9 hpf) were fixed with 4% paraformaldehyde (PFA). Dead cells within the embryos were detected using *In Situ* Cell Death Detection Kit, POD (Roche, Mannheim, Germany), and the signals were amplified using an Alexa Fluor 647-Tyramide Signal Amplification Kit (Invitrogen, Carlsbad, CA, USA) following the manufacturer’s instructions. To visualize the location of GFP-positive endoderm cells within the embryos after TUNEL, immunofluorescence analyses were performed as described [20]. Anti-GFP (Chicken antibodies, IgY fraction) (aves, Tigard, OR, USA) and CF488A goat anti-chicken IgY (Biotium, Hayward, CA, USA) were used.

### 3.4. BrdU Labeling and Detection

About 1 nL of 25 mM BrdU (Sigma, St. Louis, MO, USA) was injected into the yolk of *Tg* [*sox17:GFP*] embryos at shield stage (6 hpf). After 3 h incubation, embryos were fixed with 4% PFA. After immunofluorescence analyses for GFP, embryos were treated with 5 μg/mL of protenase K (Roche, Mannheim, Germany) for 5 min, washed with PBSDT (1% DMSO, 0.1% TritonX100 in PBS) and fixed with 4% PFA. Re-fixed embryos were treated with 2 N HCl for 20 min, treated with 0.1 M Boric acid for 10 min, washed with PBSDT and blocked with 2% FBS in PBSDT (blocking buffer) for at least 30 min. The embryos were incubated with rat anti-BrdU antibody (AbD Serotec, Oxford, UK) in blocking buffer for at least 16 h at 4 °C. After extensive washing with PBSDT, embryos were incubated with CF647 donkey anti-rat IgG (Biotium, Hayward, CA, USA) in blocking buffer for 16 h at 4 °C. Embryos were extensively washed with PBSDT and fixed with 4% PFA.

### 3.5. Imaging of Fluorescence Signals

Embryos were embedded in 1% low-melt agarose. Time-lapse image acquisition was performed with an LSM710 confocal microscope and Zen software (Zeiss, Oberkochen, Germany). Immunefluorescence signals in fixed embryos or GFP signals in live embryos were visualized and photographed using an SZX12 stereo microscope (Olympus, Tokyo, Japan), LSM710 or LSM-Duo confocal microscopes (Zeiss, Oberkochen, Germany).

### 3.6. RNA Immunoprecipitation (RIP) Assay

In accordance with the manufacturer’s protocol of the RIP-Assay Kit (MBL), complexes which consist of mRNAs and Celf1 were isolated from uninjected embryos at 9–10 hpf by using rabbit anti-Celf1 antiserum (a kind gift from Dr. Kunio Inoue). Normal rabbit serum (Thermo Scientific, Waltham, MA, USA) was used as a control. *In vivo* interaction between Celf1 and several mRNAs was tested by RT-PCR with gene-specific primers. Signal intensity was quantified using Image J software (NIH: Bethesda, MD, USA).

The sequences of gene-specific primers were as follows:

*gata5*-F: 5′-TGGTGTGCTCTGTCTCAAGC-3′;
*gata5*-R: 5′-GCTTTCCTAAGCACCGTCTG-3′;
*cdc42*-F: 5′-GACAGTAGCCCTGTAAATGGTTG-3′;
*cdc42*-R: 5′-GTTAGAAAGTTCCCTGCTTGAGAG-3′;
*ccna1*-F: 5′-CTCCCACAATCCACCAGTTT-3′;
*ccna1*-R: 5′-AATCACAGCCAGAGAGTAACCAG-3′.

### 3.7. Quantitative PCR (qPCR)

Embryos injected with control-MO or *celf1*-MOs were grown to 10 hpf. Total RNAs of embryos were isolated using Sepasol RNA I (Nacalai Tesque, Kyoto, Japan). First-strand cDNAs were synthesized from total RNA with SuperScript II (Invitrogen, Carlsbad, CA, USA) and oligo-dT primers (Invitrogen, Carlsbad, CA, USA). Quantitative real-time PCR using gene-specific primers (see above) was performed in LightCycler 480 system II (Roche, Mannheim, Germany) using KAPA SYBR FAST qPCR Kit Master Mix (KAPABIOSYSTEMS, Boston, MA, USA).

### 3.8. Statistics

An ANOVA followed by Scheffe’s test was used for comparisons of three or four groups, and a Student’s *t* test for comparisons of two groups. Results were presented as the mean ± SD, and considered significant when *p* < 0.05.

## 4. Conclusions

RNA-binding proteins control gene expression at post-transcriptional levels by binding to numerous and diverse mRNAs. A RNA-binding protein named Celf1 was characterized in organisms ranging from humans to flies. In the present study, we used zebrafish as a model system and revealed a novel role of Celf1 during early vertebrate development. We provided the evidence that Celf1 is involved in endoderm proliferation and migration, and we proposed a possible mechanism in which Celf1-dependent regulation of *gata5* and *cdc42* is required for proper formation of endoderm-derived organs during zebrafish development.

## Figures and Tables

**Table 1:** Frequency of cell death and division.

Embryo	Cell death (times/h)	Cell division (times/h)
control (*n* = 3)	0.11 ± 0.16	27.77 ± 3.72
*celf1* KD (*n* = 4)	0 ± 0	17.00 ± 3.32 *

Statistically significant difference (* *p* < 0.05) could be seen in control *vs. celf1* KD embryos.

## Supplementary Files

Supplementary File 1 Supplementary (ZIP, 27778 KB)
