# Supplementary material for: Celf1 Is Required for Formation of Endoderm-Derived Organs in Zebrafish
Source: Int J Mol Sci. 2013 Sep 3;14(9):18009–23. doi: 10.3390/ijms140918009 (PMC3794766; doi:10.3390/ijms140918009)
Supplement: Supplementary File 1 — Supplementary (ZIP, 27778 KB) [file ijms-14-18009-s001.zip › ijms-37472-final-suppl/ijms-37472-final-suppl..pdf]

Supplementary Information

Movie Legends

Movie 1. Behavior of endoderm cells in the control embryo. The migration and division of endoderm cells in the control embryo during 7–10 hpf. The cell position was determined every three minutes. Dividing cells were marked by red.
Movie 2. Behavior of endoderm cells in the *celf1* KD embryo. The migration and division of endoderm cells in the *celf1* KD embryo during 7–10 hpf. The cell position was determined every three minutes. Dividing cells were marked by red.

© 2013 by the authors; licensee MDPI, Basel, Switzerland. This article is an open access article distributed under the terms and conditions of the Creative Commons Attribution license (http://creativecommons.org/licenses/by/3.0/).
